# MRCKα Is a Suppressor of GEF-H1/RhoA/MRTF Signaling in Tubular Cells

**DOI:** 10.3390/cells15050447

**Published:** 2026-03-02

**Authors:** Veroni S. Sri Theivakadadcham, Qinghong Dan, Brian Wu, Shruthi Venugopal, Vida Maksimoska, Aysegul Yucel-Polat, Andras Kapus, Katalin Szászi

**Affiliations:** 1 Keenan Research Centre for Biomedical Science of the St. Michael’s Hospital, Unity Health Toronto, Toronto, ON M5B 1T8, Canada; veronisaratha@hotmail.com (V.S.S.T.); qinghong.dan@unityhealth.to (Q.D.); vida.maksimoska@mail.utoronto.ca (V.M.); andras.kapus@unityhealth.to (A.K.); 2 Department of Medicine, Queen’s University, Kingston, ON K7L 3N6, Canada; b.wu@queensu.ca; 3 Department of Surgery, University of Toronto, Toronto, ON M5T 1P5, Canada; shruthi.venugopal2@unityhealth.to (S.V.); aysegul.yucel-polat@unityhealth.to (A.Y.-P.)

**Keywords:** kidney fibrosis, tubular cells, RhoA signaling, guanine nucleotide exchange factor, *ARHGEF2* (GEF-H1), myocardin-related transcription factor, fibrogenic mediators, Myotonic Dystrophy Kinase-related Cdc42-binding kinase α

## Abstract

**Highlights:**

**What are the main findings?**

- Myotonic Dystrophy Kinase-related Cdc42-binding kinase (MRCK)α is a new interactor of GEF-H1 that suppresses GEF-H1/RhoA signaling and modulates actin remodeling and phospho-MLC in tubular cells.
- MRCKα is a suppressor of MRTF-dependent fibrotic genes in tubular cells, and its expression is reduced in a kidney fibrosis mouse model.

**What are the implications of the main findings?**

- MRCKα may exert crucial negative feedback on stimulus-induced GEF-H1 activation to suppress GEF-H1/RhoA signaling.
- Our study implicates MRCKα as a potential new suppressor of tubular reprogramming in kidney fibrosis.

**Abstract:**

Tubule-derived pro-fibrotic mediators are central for the development of kidney fibrosis. We previously showed that fibrotic stimuli activate and elevate GEF-H1 (*ARHGEF2*) in tubular cells, leading to RhoA-dependent fibrotic reprogramming. In search of new mechanisms of GEF-H1 regulation, here we used immunoprecipitation and proximity ligation assay to show interaction between GEF-H1 and Myotonic Dystrophy Kinase-related Cdc42-binding kinase (MRCK)α in tubular cells. MRCKα silencing elevated GEF-H1 activity, and induced GEF-H1-dependent RhoA activation, stress fibre formation and myosin light chain phosphorylation. MRCKα depletion also elevated phospho-cofilin levels in a RhoA-dependent manner. The fibrogenic cytokine TGFβ1 rapidly increased binding between GEF-H1 and MRCKα, while MRCKα silencing augmented TGFβ1-induced GEF-H1 activation, suggesting a negative feedback loop. An mRNA array detecting fibrogenic genes revealed increase in a subset of basal and TGFβ1-induced genes following MRCKα depletion. MRCKα silencing promoted nuclear translocation of the profibrotic transcriptional co-activator Myocardin-Related Transcription Factor (MRTF), and MRTF-A+B depletion prevented increase in *ACTA2* (α-smooth muscle actin), a key marker of fibrotic reprogramming. Finally, total MRCKα mRNA was reduced in a murine kidney fibrosis model, and immunohistochemistry revealed a decrease in tubular MRCKα. Taken together, we identified MRCKα as a new suppressor of GEF-H1/RhoA/MRTF signaling. Reduced MRCKα expression in kidney fibrosis may promote tubular fibrotic gene expression.

## 1. Introduction

Chronic kidney disease (CKD), a common consequence of diabetes and high blood pressure is characterized by the gradual loss of kidney functions. The underlying pathology is hallmarked by chronic inflammation and tubulointerstitial fibrosis, i.e., excessive deposition of extracellular matrix (ECM) that destroys functional tissue [1]. The number of patients with CKD and kidney fibrosis is rapidly increasing, leading to a serious public health problem [2]. However, our understanding of the molecular pathogenesis of fibrosis remains incomplete. Activated fibroblasts and myofibroblasts that overproduce ECM are key pathogenic executors in fibrosis. Accumulating evidence reveals the importance of cues derived from surrounding cells, including the tubular epithelium, in activation of these ECM producing cells (reviewed in [3,4,5]). Epithelial-mesenchymal crosstalk is mediated by factors released from tubular cells that undergo a pro-fibrotic phenotypic reprogramming, or partial epithelial-mesenchymal transition, leading to a pro-fibrotic epithelial phenotype (PEP) [6]. This process can be induced by injury and cytokines, including TNFα and TGFβ1 [7,8,9]). TGFβ1 is one of the most potent inducers of fibrogenesis (e.g., [10]). We and others have highlighted the significance of RhoA signaling in tubular pro-fibrotic reprogramming [6,11,12]). Our data revealed early RhoA activation in tubular cells in a murine model of kidney injury and fibrosis [6] and in cultured cells following stimulation by inflammatory mediators [11,13]. Myocardin-related Transcription Factors (MRTF-A and B) are key co-activators of serum response factor (SRF) that act downstream from RhoA (reviewed in [14,15]). MRTFs shuttle between the cytosol and the nucleus, controlled by the polymerization state of actin. RhoA-induced F-actin polymerization stimulates their nuclear accumulation, where the MRTF/SRF complex binds to CARG domains in the promoters of fibrosis-related target genes and initiates their transcription [16,17]. Key MRTF-dependent tubule-derived fibrogenic mediators include Cellular communication network factor 2 (CCN2), also known as Connective Tissue Growth Factor (CTGF) and TGFβ family proteins [6].

RhoA dysregulation during inflammation and injury contributes to a vicious cycle that can lead to maladaptive repair and fibrosis [15,18]. Rho family proteins are controlled by a large network of guanine nucleotide exchange factors (GEF) that promote their activation, and GTPase activating proteins (GAP) that enhance inactivation [19,20]. We have identified GEF-H1 (*ARHGEF2*), as a key activator of RhoA in kidney tubular cells [13]. GEF-H1 is a Dbl family GEF highly expressed in epithelial cells [21,22]. Its dysregulation was associated with various pathological conditions, including cancer, organ fibrosis and kidney disease [12,23,24,25,26]. We have recently shown that GEF-H1 expression is elevated in a mouse kidney fibrosis model and in tubular cells stimulated by pro-fibrotic cytokines [11]. Thus, evidence points to a central role of GEF-H1 in fibrogenesis. GEF-H1 is controlled by interacting proteins that localize it to the microtubules and intercellular junctions, and by phosphorylation (e.g., [27,28,29,30]). It is activated in response to an array of stimuli, including various inflammatory and fibrogenic input, such as TGFβ1, TNFα and mechanical stress [13,26,31,32]. However, fibrosis-relevant control of GEF-H1 activity remains incompletely understood. Here we show that Myotonic dystrophy-related Cdc42-binding kinase α (MRCKα) is an interactor of GEF-H1. MRCK belongs to the AGC (PKA, PKG and PKC) kinase family [33,34,35,36]. It has three isoforms, closely related to Rho kinase. MRCK family kinases control epithelial apico-basal polarization, cell migration and tissue remodeling [35]. However, their role in organ fibrosis or kidney disease remains unexplored.

In this study we show that MRCKα inhibits GEF-H1/RhoA signaling, thereby reducing cytoskeleton remodelling, MRTF nuclear translocation and MRTF-dependent fibrotic gene expression. Overall, our findings establish MRCKα as a new suppressor of the GEF-H1/RhoA/MRTF axis in tubular cells, with important implications for fibrogenesis.

## 2. Materials and Methods

### 2.1. Reagents and Antibodies

TGFβ1 was from MedChem Express (Monmouth Junction, NJ, USA, Cat#HY-P7118) or R&D Systems (Minneapolis, MN, USA, Cat#240-B-002). Rhosin Hydrochloride (Cat#5003) was from Tocris Bioscience (Biotechne), Abingdon, UK.

The following GEF-H1 antibodies were used: for immunoprecipitation: Cat# NBP2-21577, RRID: AB_3265603 (Novus, Centennial, CO, USA); for proximity ligation assay, mouse anti-GEF-H1 (Thermo Fisher Scientific, Waltham, MA, USA) Cat# MA5-27803, RRID:AB_2735201) and for Western blotting: Cat# 4076, RRID: AB_2060032 (Cell Signaling Technologies, Danvers, MA, USA). MRCKα antibodies used were as follows: for immunoprecipitation and Western blotting Cat# A302-694A, RRID:AB_10750425 (Thermo Fisher Scientific), and Cat#81681, RRID: AB_3718698 (Cell Signalling Technology); for proximity ligation assay and immunohistochemistry: rabbit anti-MRCKα Cat#GTX10259 RRID:AB_1240609 (GeneTex Inc., Irvine, CA, USA). The following antibodies were obtained from Cell Signaling Technology: RhoA (Cat# 2117S, RRID: AB_10693922), Phospho-Myosin Light Chain 2 (Thr18/Ser19) (E2J8F0) (Cat# 95777, RRID:AB_3677547), p-cofilin (Ser3) (Cat# 3313, RRID: AB_2080597), cofilin (Cat# 5175, RRID: AB_10622000), MRTF B (Cat# 14613, RRID: AB_2798539). Other antibodies used were: GFP (Cat# 66002-1-Ig, RRID: AB_11182611) and MRTF-A (Cat# 21166-1-AP, RRID:AB_2878822) from Proteintech (Rosemont, IL, USA); GAPDH (Cat# 39-8600, RRID: AB_2533438) from Thermo Fisher Scientific, α-smooth muscle actin (SMA) (Cat# ab150301, RRID:AB_3675461) from Abcam (Cambridge, MA, USA) and MRCKβ antibody (Cat# PA5-99655, RRID:AB_2818588) from Thermo Fisher Scientific. Secondary antibodies used for Western blotting were from Cell Signaling Technology: HRP-linked anti-rabbit IgG (Cat# 7074, RRID: AB_2099233) and HRP-linked anti-mouse IgG, (Cat#7076, RRID: AB_330924). Normal Rabbit IgG was from Cell Signaling Technology (Cat# 2729, RRID:AB_1031062)

### 2.2. Cell Culture and Treatment

LLC-PK_1_, a kidney tubule epithelial cell line (male) was from the European Collection of Authenticated Cell Cultures (Wiltshire, UK), (ECACC Cat# 86121112, RRID: CVCL_0391). This cell line was used in our previous studies, e.g., [11,12]. Human Embryonic Kidney-293 (HEK-293) cells (female) (Cat# CRL-1573, RRID: CVCL_0045) were from the American Type Culture Collection (Manassas, VA, USA). Tissue culture media and reagents for culturing LLC-PK_1_ and HEK cells were from Thermo Fisher/Life Technologies. Both cell lines were maintained in a low glucose DMEM medium supplemented with 10% fetal bovine serum and 1% penicillin-streptomycin in an atmosphere containing 5% CO_2_. Unless otherwise stated, cells were serum depleted overnight before experiments. Human hTERT-immortalized renal proximal tubule epithelial cells (RPTEC/TERT1) (male) were from The American Type Culture Collection (CRL-4031). These cells were cultured in Minimum Essential Medium Eagle (Cat# M4526, Millipore/Sigma-Aldrich, Burlington, ON, Canada), with RPTEC Complete supplement (Cat# MTOXRCSUP) and Gentamicin (G1397). RPTECs were incubated in culture medium without the supplement overnight prior to experiments.

For TGFβ1 treatment, cells were grown to 100% confluence, serum/supplement depleted as indicated, and TGFβ1 (10 ng/mL) was added for the indicated times.

### 2.3. Plasmid and Short Interfering RNA (siRNA) Transfection

GFP-GEF-H1-WT or GFP-GEF-H1-ΔC constructs were a kind gift from Dr. M. Kohno (Nagayo, Nagasaki, Japan). These constructs were generated as described in [37] by cloning into a pEGFP-C1 expression vector. Sequencing verified that the GFP-tagged construct encodes isoform 2 of GEF-H1. MRCKα (*CDC42BPA*) cloned into the pReceiver-M56 vector containing an mCherry tag was purchased from GeneCopoeia (Rockville, MD, USA Cat#EX-T0588-M56), and pcDNA3-EGFP was from Addgene (plasmid #13031).

For immunoprecipitation experiments HEK-293 cells were grown in 10 cm dishes to 70–80% confluence and transfected with 4–5 µg of GFP alone, or GFP-tagged GEF-H1 constructs using Fugene 6 transfection reagent (Cat# E2691, Promega Madison, WI, USA) following the manufacturer’s instructions. Cells were used 24 or 48 h post-transfection.

For siRNA-mediated silencing oligonucleotides were purchased from Dharmacon/Horizon Discovery (Lafayette, CO, USA). The non-related control siRNA (NR siRNA) was purchased from Dharmacon/Horizon Discovery (Cat nr D-001810-01-50) or MedChemExpress (Cat#HY-150150). Table 1 lists the siRNA sequences used in LLC-PK_1_ cells. For silencing proteins in human RPTEC/hTERT cells, the following predesigned siRNAs were used: ON-TARGETplus Human *CDC42BPA* (8476) siRNA cat# J-003814-14 (MRCKα siRNA#1) and J-003814-15 (MRCKα siRNA#2) (Horizon Discovery) and human *ARHGEF2* siRNA (9181) cat #J-009883-06 and #J-009883-07.

Cells were transfected using 100 nM siRNA using Lipofectamine RNAiMAX transfection reagent (Cat#13778075, Thermo Fisher Scientific) following the manufacturer’s instructions. For MRTF-A and B silencing, 50 nM of each siRNA was used. Unless otherwise indicated, the experiments were performed 48 h after transfection. For co-silencing GEF-H1 and MRCKα, the cells were sequentially transfected, as follows. First, they were transfected with GEF-H1 siRNA, and 24 h or 6 h later (as indicated in the legends), they were transfected with MRCKα siRNA for an additional 24 h. Downregulation was routinely verified using Western blotting.

### 2.4. Immunoprecipitation (IP)

LLC-PK_1_, HEK-293 or RPTEC/hTERT cells were grown in 10 cm dishes. Where indicated, cells were transfected as described above. Cells were grown to 100% confluency, washed with ice-cold PBS and lysed with ice-cold NP-40 lysis buffer (150 mM NaCl, 30 mM HEPES, 1% NP40, 0.25% Sodium deoxycholate, PH 7.5) containing 1 mM Na_3_VO_4_ (Cat#0758, New England Biolabs, Whitby, ON, Canada), 1 mM phenylmethylsulphonyl fluoride (Cat# PMS444, Bioshop Burlington, ON Canada), protease and phosphatase inhibitors (cOmplete Mini, EDTA-free (cat# 4693159001) and PhosSTOP (cat# 4906845001) from Roche Diagnostics, Basel, Switzerland). Lysates were centrifuged, and an aliquot of the supernatant was retained as input sample. The remaining supernatants were used for pre-clearing. For precipitating endogenous proteins, Pierce™ Protein A/G Agarose beads (Cat#PI20421, Thermo Fisher Scientific) were blocked by incubating with 2% bovine serum albumin (BSA) in NP40 lysis buffer for 1 h at 4 °C in a rotator, followed by washes with NP40 lysis buffer. The supernatants from the cell lysates were precleared for 1 h at 4 °C in a rotator using the A/G Agarose beads, then incubated with 3 µg of GEF-H1, or MRCKα antibody (1 h), followed by incubation with blocked Pierce™ Protein A/G Agarose beads (3 h). Beads without primary antibody, or with normal rabbit IgG were used as control, as indicated.

For precipitating GFP or GFP-tagged GEF-H1, lysates were precleared using ChromoTek Binding Control Agarose Beads (Cat# bab, Proteintech), and immunoprecipitation was performed using ChromoTek GFP-Trap^®^ Agarose (cat#:gta, RRID:AB_2631357) from Proteintech, for 3 h at 4 °C using a rotator.

For all IPs, pelleted beads were washed with ice-cold NP-40 lysis buffer three times, resuspended and boiled in Laemmli Sample Buffer (Bio-Rad, Hercules, CA, USA, Cat# 1610737) and analysed by Western blotting.

### 2.5. Western Blotting

Western blotting was done as in our previous studies (e.g., [11]). Briefly, following the indicated treatments, cells were washed, lysed with ice-cold lysis buffer (100 mM NaCl, 30 mM HEPES pH7.5, 20 mM NaF, 1 mM EGTA, 1% Triton X-100) supplemented with 1 mM Na_3_VO_4_, 1 mM phenylmethylsulphonyl fluoride, and protease and phosphatase inhibitors. Lysates were centrifuged and protein concentration determined using bicinchoninic acid assay (Thermo Fisher Scientific/Pierce Biotechnology). Proteins were separated by SDS polyacrylamide gel electrophoresis and transferred to nitrocellulose membrane using standard protocols. The blots were blocked in Tris-buffered saline with Tween (TBST) containing 3% BSA for 1 h, then incubated with the indicated primary antibodies overnight at 4 °C, followed by washes, incubation with the corresponding peroxidase-conjugated secondary antibodies and visualization with the enhanced chemiluminescence (ECL) method (Cat#1705060, Bio-Rad, Hercules, CA, USA). For multiple probing of the same blot, the blot was either stripped and re-probed, or the membrane was cut horizontally and probed with different primary antibodies. ECL signals were captured using a BioRad ChemiDoc Imaging system. Densitometry was performed using ImageLab 6.1. Values were normalized using GAPDH as loading control, and expressed as fold change from control, taken as unity.

### 2.6. GEF-H1 and RhoA Activation Assay

Active GEF-H1 and RhoA (GTP-bound) were captured from cell lysates using GST-RhoA(G17A) or GST-RhoA-binding domain (RBD), amino acids 7–89 of Rhotekin, respectively, as described in [13,38]. RhoA(G17A) cannot bind nucleotide and therefore has high affinity for activated GEFs [39]. Confluent LLC-PK_1_ or RPTEC/hTERT cells were lysed with ice-cold assay buffer (100 mM NaCl, 50 mM Tris base (pH 7.6), 20 mM NaF, 10 mM MgCl_2_, 1% Triton X-100, 0.5% deoxycholic acid, 0.1% SDS) containing 1 mM Na_3_VO_4_ and cOmplete Mini, EDTA-free protease inhibitor. After centrifugation, an aliquot of the supernatant was retained (total GEF-H1 or RhoA). The remaining supernatants were incubated with 20–25 µg of GST-RhoA(G17A) or GST-RBD beads (45 min, at 4 °C), followed by extensive washes. Total and captured (active) proteins were analyzed by Western blotting and quantified by densitometry, as previously described [11]. Precipitated (active) GEF-H1 or RhoA for each condition was normalized using the corresponding total protein, and the fold change relative to control was calculated.

### 2.7. Immunofluorescence Microscopy

LLC-PK_1_ cells grown on coverslips were transfected and treated as indicated. Cells were then fixed with 4% paraformaldehyde, washed with PBS, permeabilized and quenched with 0.1% Triton-PBS containing 100 mM glycine. Following washing, coverslips were blocked with 3% BSA in PBS, then incubated with primary antibody (1:100) for 1 h. Next, coverslips were washed and incubated with Alexa Fluor^®^ 488-conjugated anti-rabbit IgG (H+L), F(ab’)2 Fragment secondary antibody (1:1000, Cell Signaling Technology, Cat# 4413, RRID: AB_10694110) and 4′,6-Diamidine-2′-phenylindole dihydrochloride (DAPI) (1:1000) (Cat# 10236276001, Roche Diagnostics). For F-actin staining, cells were incubated with Alexa Fluor^®^ 488 Phalloidin (Cat# 8878, Cell Signaling Technology) for 30 min along with DAPI. The slides were visualized either using a Zeiss Widefield Microscope (White Plains, NY, USA) (63× objective), or a WaveFX spinning-disk confocal microscope (Quorum Technologies, Guelph, ON, Canada) with an ORCA-flash4.0 digital camera with Gen II sCMOS image sensor. For confocal images, maximum intensity projection pictures were generated from Z-stack using the Metamorph software 7.8. All parameters for image acquisition were kept constant. Control and treatment groups were processed in parallel at the same time under the same conditions.

Image analysis was performed using the ImageJ-FIJI software Version 1.54p [40]. Images were separated into their individual fluorescence channels. To quantify fluorescence intensity of the F-actin or pMLC staining, first the background was adjusted using a Gaussian blur filter with a sigma value of 2 pixels, and a uniform threshold value of 1400. Next, regions of interest (ROIs) corresponding to the tissue border were manually delineated, and the mean fluorescence intensity was measured within each ROI.

To quantify nuclear-to-cytoplasmic (N/C) signal ratios of MRTF-A, nuclei were segmented using a threshold and nuclear ROIs were generated using DAPI staining. The target-protein channel was duplicated allowing independent assessment of nuclear and cytoplasmic signals. For nuclear intensity measurements the non-nuclear signal was cleared, while for cytoplasmic measurements the nuclear signal was cleared. Integrated fluorescence intensities were then recorded for both the nuclear and cytoplasmic compartment, and the nuclear/cytoplasmic (N/C) ratio was calculated as nuclear intensity divided by cytoplasmic intensity using the same ROI sizes.

### 2.8. Proximity Ligation Assay (PLA)

RPTEC/hTERT cells grown on coverslips were fixed, washed and permeabilized as described in 2.7. The PLA assay was performed using the Duolink In Situ Orange kit (cat#DUO92102, Millipore/Sigma-Aldrich), as recommended by the manufacturer. Briefly, cells were blocked with the Duolink Blocking Solution (1 h, room temperature). Primary antibodies (mouse anti-GEF-H1 (1:50) and rabbit anti-MRCKα (1:500)) were diluted in the antibody diluent and incubated in a humidified chamber overnight at 4 °C. Following washing, the PLA probes (Duolink In Situ PLA Probe Anti-Mouse MINUS and Anti-Rabbit PLUS, 1:5 dilution in antibody diluent) were added for 1 h in a pre-heated humidified chamber at 37 °C. Ligation was performed using Duolink Ligase (1:40 in ligation buffer, 30 min) at 37 °C. The amplification reaction was performed using Duolink Polymerase (1:80, 100 min, 37 °C). To simultaneously visualize F-actin, following amplification, cells were thoroughly washed, then stained with Alexa Fluor^®^ 488 Phalloidin for 30 min. Cells were mounted using the Duolink In Situ Mounting Medium containing DAPI. Images were taken using a WaveFX spinning-disk confocal microscope (Quorum Technologies) with an ORCA-flash4.0 digital camera with Gen II sCMOS image sensor. (63× objective). Maximum intensity projection pictures were generated from Z-stack using the Metamorph software. The PLA signal was quantified by counting the puncta in each microscopic field and dividing it by the number of nuclei. For each experiment 3–5 randomly selected fields/coverslips were counted.

### 2.9. RT2 Profiler Fibrosis Array

An RT^2^ Profiler™ Pig Fibrosis PCR Array was used to determine levels of 84 fibrosis-related genes (Cat# 330231, GeneGlobe ID: PASS-120Z, Qiagen, Montreal, QC, Canada). LLC-PK_1_ cells were transfected with 100 nM NR or MRCKα siRNA. 24 h post-transfection, the medium was replaced with serum-free DMEM medium followed by treatment with 10 ng/mL TGFβ1 for 24 h. RNA was extracted using the RNeasy Mini Kit (Cat# 74104, Qiagen) and 1.5 µg of RNA was used for gDNA elimination and cDNA synthesis using the RT^2^ First strand kit (Cat# 330401, Qiagen). A stock solution for each tested condition was prepared using the RT^2^ SYBR Green ROX qPCR Mastermix (Cat# 330523, Qiagen), plated in the RT^2^ Profiler™ PCR Array and PCR was performed using a QuantStudio™ 7 Flex Real-Time PCR System (Thermo Fisher Scientific). Relative gene expression was calculated using the geometric mean of the loading controls provided with the array, with the ∆∆Ct method. Either fold changes from control, or normalized 2^−∆Ct^ values were graphed, as indicated in the figures. Fold changes were inputted into the R Studio Statistical software (version 4.4.2, R core team 2023, and Heatmap R package version 1.0.12, “pheatmap” plugin, https://CRAN.R-project.org/package=pheatmap, accessed on 10 January 2025). Heatmaps show Z scores (indicating the number of standard deviations a data point is from the mean) of genes across treatment groups. Gene expression values underwent hierarchical clustering according to the pheatmap package. 

### 2.10. RNA Extraction and RT-PCR

LLC-PK_1_ cells were transfected with the indicated siRNAs. Where indicated, 24 h post-transfection, the medium was replaced with serum-free DMEM medium and treated with 10 ng/mL TGFβ1 for 24 h. For both LLC-PK_1_ cells and mouse tissue, total RNA was extracted using the RNeasy Mini Kit (Qiagen) and 1 µg of RNA was converted to cDNA using iScript™ cDNA Synthesis Kit (Bio-Rad, Cat# 1708890) following the manufacturer’s instruction. SYBR green-based real-time PCR was performed using QuantStudio™ 7 Flex Real-Time PCR System. Peptidylprolyl isomerase A (PPIA) and GAPDH were used as the reference gene for the LLC-PK_1_ cells and the mouse tissue, respectively. Relative gene expression was calculated using the 2^−∆Ct^ method. Table 2 lists the qPCR Primer sequences used for LLC-PK_1_ cells, and Table 3 lists the primers used for the mouse kidney tissue.

### 2.11. Unilateral Ureteral Obstruction (UUO) Mouse Model

UUO was performed as described in [6]. All procedures were approved by the St. Michael’s Hospital animal care committee (#ACC541, approved 24 June 2014). Animals were included based on their age. The left ureters of 6–8-week-old male C57BL/6 mice (5/group) were ligated. Sham controls underwent surgery, but the ureters were left undisturbed. No animals were lost during the procedure or post-surgery. After sacrificing mice at seven days post-surgery, the left kidneys were collected. Half of the kidneys were snap-frozen, the other half was fixed in formalin, dehydrated, cleared, embedded in paraffin, and stored for use for histology. The frozen tissues were homogenized and processed for RNA preparation. Samples were stored in −80 °C.

### 2.12. Immunohistochemistry

Paraffin-embedded kidney sections from sham and UUO-operated mice (7-days post-surgery, *n* = 3, randomly selected) were deparaffinized in xylene followed by rehydration using a graded series of alcohol washes. Antigen retrieval was performed by boiling sections in 10 mM sodium citrate (pH 6.0) (20 min). Endogenous peroxide was blocked by 3% H_2_O_2_. Nonspecific IgG binding was blocked by incubating sections with 2% goat serum for 1 h (Vector Laboratories, Newark, CA, USA; MP-745115), followed by incubation with MRCKα primary antibody (GeneTex, Irvine, CA, USA, Cat. No. GTX102598, 1:100, overnight at 4 °C). Sections were incubated with ImmPRESS HRP goat anti-rabbit IgG polymer detection kit (Vector Laboratories; SK-4105) (30 min), and counterstained with Surgipath SelecTech (Richmond, IL, USA) Hematoxylin 560 as per manufacturer’s instructions. Histological slides were imaged on a Zeiss Axioscan.Z1 slide scanner, using a 10× objective. For exporting the images, a 10% digital export zoom was used. DAB immunostaining was quantified in ImageJ/FIJI (version 1.54r) [40] using the H–DAB color deconvolution tool. For each kidney section, 4–5 representative regions were exported as uncompressed TIFF format with identical display settings, ensuring complete coverage of the tissue. Areas containing the tissue in the image were selected. A uniform threshold was applied to the DAB channel (Colour_2) using a fixed range (0–130) determined from the histogram. The percentage of DAB-positive area (%Area) was measured in 4–5 ROI/sample and the data were expressed as mean.

### 2.13. Statistical Analysis

Western blot and immunofluorescent pictures shown are representatives of at least three independent experiments. The graphs were generated using GraphPad Prism software (version 10.6.1) and the data are presented as means ± S.D. of the number of independent experiments indicated in the figure legends (n). Statistical analysis was performed using GraphPad Prism. Data normalized to the control were analysed using Welch’s *t*-test (for 2 samples) and significance was depicted using * symbols, or one-way ANOVA with Šidak’s multiple comparisons test (depicted with #), as indicated in the legends. Non-normalized samples were compared using unpaired *t*-test (denoted by *) or one-way ANOVA, with Tukey’s post test. Significance for ANOVA was denoted using #. Significance indicated on the figures is as follows: one symbol (# or *) *p* < 0.05; two symbols (## or **) *p* < 0.01; three symbols (### or ***) *p* < 0.001; four symbols (#### or ****) *p* < 0.0001.

## 3. Results

### 3.1. Association Between MRCKα and the N-Terminus of GEF-H1

**Figure 1:**
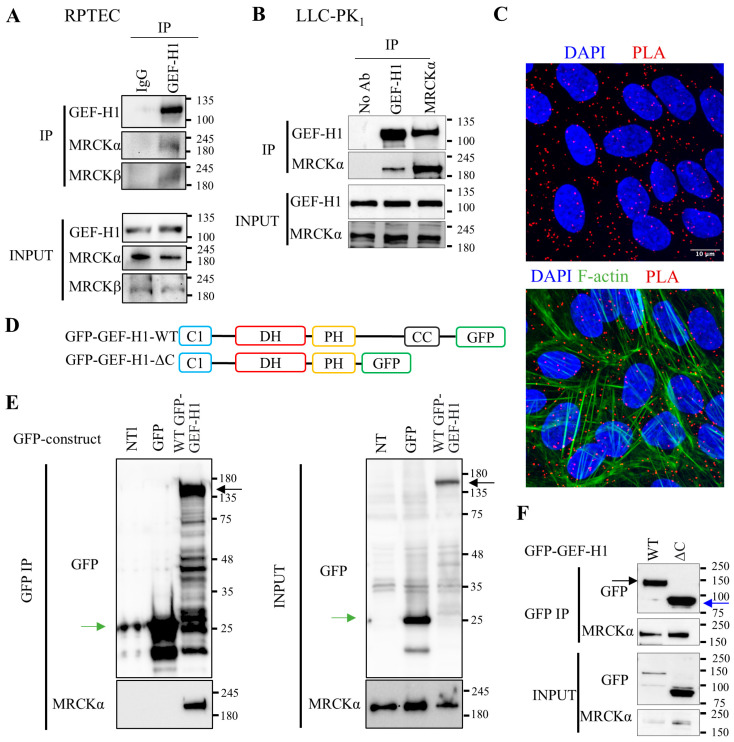
MRCKα interacts with the N-terminus of GEF-H1. (**A**) Endogenous GEF-H1 was immunoprecipitated from RPTEC/hTERT cells and co-immunoprecipitated proteins were detected by Western blotting (representative blot of *n* = 3). IgG indicates non-immune IgG plus beads (**B**). Endogenous GEF-H1 or MRCKα were immunoprecipitated from LLC-PK_1_ cells and co-precipitated proteins detected as in A. “No Ab” denotes IP without primary antibody, with beads alone. (**C**) RPTEC/hTERT cells were grown on coverslips and PLA was performed using GEF-H1 and MRCKα-specific antibodies, as described in the Methods. The nuclei were counterstained using DAPI. In the bottom picture, F-actin was visualized using Alexa Fluor^®^ 488 Phalloidin. The pictures were taken using a WaveFX spinning-disk confocal microscope (63× magnification). (**D**) GFP-tagged GEF-H1 constructs used in the experiments. Domains of GEF-H1 include C1: zinc finger-like motif; DH: Dbl-homology domain; PH: pleckstrin homology domain. CC: Coiled-coil region. (**E**,**F**) The indicated constructs were transfected in HEK-293 cells and immunoprecipitated through the GFP tag, and co-immunoprecipitation of MRCKα was visualized by Western blotting (representative blots from *n* = 3). NT: non-transfected. Arrows: green: GFP, black WT-GFP GEF-H1, blue: GFP-GEF-H1-ΔC.

In our previous studies we identified GEF-H1 as a master regulator of RhoA signalling and fibrotic reprogramming in tubular cells (e.g., [11,12]). GEF-H1 was recently shown to interact with MRCKα, although the functional relevance of this was not explored [41]. To confirm this interaction in tubular cells, we immunoprecipitated endogenous GEF-H1 from human proximal tubule cells (RPTEC/hTERT) (Figure 1A), and detected co-precipitation of MRCK using western blotting. Interestingly, both MRCKα and MRCKβ co-precipitated with GEF-H1. We verified the co-IP of GEF-H1 and MRCKα in another tubular line, LLC-PK_1_ cells, a porcine proximal tubule cell line (Figure 1B). Conversely, precipitation of endogenous MRCKα yielded well-detectable co- precipitation of GEF-H1, providing a double-sided verification of the interaction between the two proteins (Figure 1B). Adding a non-specific IgG or omitting the primary antibody during the IP resulted in no co-precipitated MRCKα or GEF-H1 (Figure 1A,B).

Next, to verify association between GEF-H1 and MRCKα, we performed in situ proximity ligation assays (PLA) in RPTEC/hTERT. We used the Duolink PLA kit to visualize proximity of the specific antibodies against GEF-H1 and MRCKα. The presence of discrete red puncta indicates that the proteins were within <40 nm (Figure 1C). Co-staining of F-actin revealed that the PLA signal indeed occurred within the cells. The signal depended on the use of the primary antibodies, as it was significantly reduced in cells transfected with an MRCKα or GEF-H1 siRNA (Figure S1). Taken together, these data verify in situ interaction between MRCKα and GEF-H1. We next asked which portion of GEF-H1 mediated the interaction with MRCKα. GEF-H1 contains a zinc finger-like motif (C1), a Dbl-homology domain (DH), a Pleckstrin homology domain (PH) and a coiled-coil (CC) region [22]. Using GFP-tagged GEF-H1 constructs expressed in HEK cells (Figure 1D), we found that the GFP-tagged WT construct, IP-ed through the GFP tag, efficiently co-precipitated MRCKα (Figure 1E,F). Importantly, the co-precipitation was specific, since MRCKα was not pulled down from non transfected or GFP-transfected cells (Figure 1E). We next compared co-precipitation of MRCKα with the full-length (WT) and a truncated GEF-H1 lacking the C-terminal part, that contained only the C1, DH and PH domains (Figure 1D). As shown in Figure 1F, MRCKα strongly co-precipitated with both the GFP-tagged full length GEF-H1 and the truncated mutant containing only the N-terminal portion (GEF-H1-ΔC), indicating that the C-terminus is not necessary for the interaction between GEF-H1 and MRCKα.

### 3.2. MRCKα Inhibits GEF-H1-Mediated RhoA Activation

**Figure 2:**
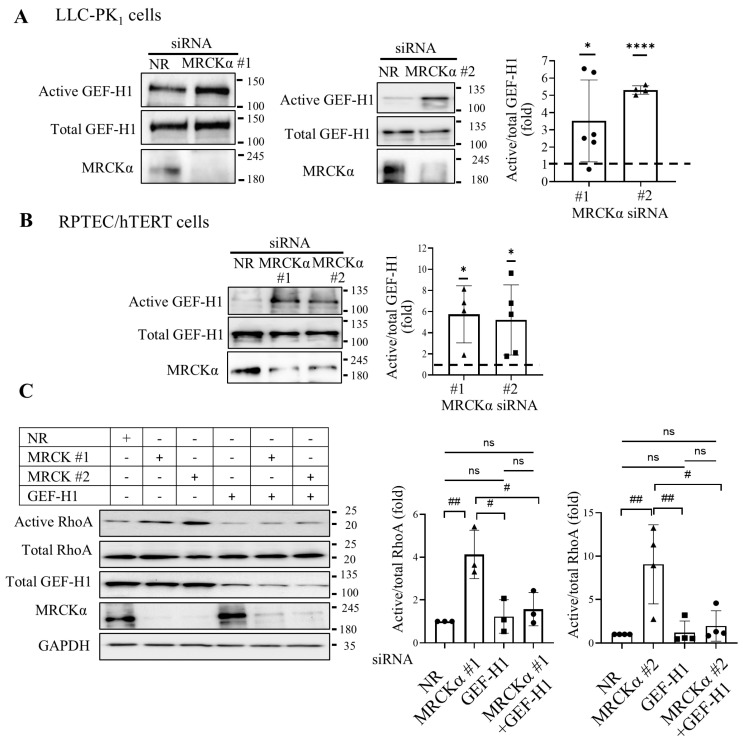
MRCKα suppresses GEF-H1 and RhoA activation. (**A**,**B**) LLC-PK_1_ (**A**) or RPTEC/hTERT cells (**B**) were transfected with non-related (NR) siRNA, or two different MRCKα-specific siRNAs (100 nM, 48 h) per species, corresponding to the porcine (**A**) or the human (**B**) sequences. The cells were lysed and active GEF-H1 was captured using GST-RhoA(G17A) beads. Precipitated (active) and total GEF-H1 were detected by Western blotting and quantified by densitometry. The graphs show results obtained with the different siRNAs. Active GEF-H1 was normalized to the corresponding total GEF-H1 and expressed as fold change from control (taken as 1, indicated by the dotted line). In (**A**) siRNA #1 *n* = 6; siRNA #2: *n* = 4. In (**B**) siRNA #1 *n* = 4; siRNA #2: *n* = 5. * *p* < 0.05, **** *p* < 0.0001). Welch’s *t* test vs. control). (**C**) LLC-PK_1_ cells were transfected with NR, MRCKα (#1 or #2) or GEF-H1-specific siRNAs or their combination, as indicated. Active RhoA was captured using GST–RBD-coupled beads. The precipitated (active) and total RhoA were detected by Western blotting. GEF-H1, MRCKα and GAPDH in the total cell lysates were also detected. Active RhoA was normalized to the corresponding total RhoA, and expressed as fold change from control (taken as 1). For MRCKα siRNA #1 *n* = 3, siRNA #2 *n* = 4, # *p* < 0.05 and ## *p* < 0.01, one way ANOVA with Šídák’s multiple-comparison test vs. the indicated condition. ns: non-significant.

Having verified the interaction between MRCKα and GEF-H1, we next asked if MRCKα influenced GEF-H1 activity. We silenced MRCKα in LLC-PK_1_ cells (Figure 2A) or in human RPTEC/hTERT cells (Figure 2B), using two independent MRCKα-specific siRNAs in each cell line. All siRNAs potently reduced MRCKα expression. GEF-H1 activity was detected using affinity precipitation with GST-RhoA(G17A), a mutant with high affinity for activated GEFs, as in our earlier studies (e.g., [13]). Silencing MRCKα resulted in a significant augmentation of GEF-H1 activity in both cell lines (Figure 2A,B). The two siRNAs yielded similar results in both cell lines, corroborating the finding and verifying that this effect was indeed due to MRCKα depletion. These data strongly suggest that in resting cells, MRCKα is an inhibitor of GEF-H1.

We next asked whether MRCKα silencing-induced GEF-H1 activation was sufficient to promote RhoA activation. The GST-RBD affinity precipitation assay revealed that MRCKα silencing using two independent siRNAs indeed significantly increased RhoA activity (Figure 2C). Importantly, co-silencing GEF-H1 prevented RhoA activation induced by MRCKα downregulation, indicating that RhoA activation was mediated by GEF-H1.

### 3.3. MRCKα Silencing Augments GEF-H1 Dependent Stress Fiber Formation and MLC Phosphorylation, and RhoA-Dependent Cofilin Phosphorylation

**Figure 3:**
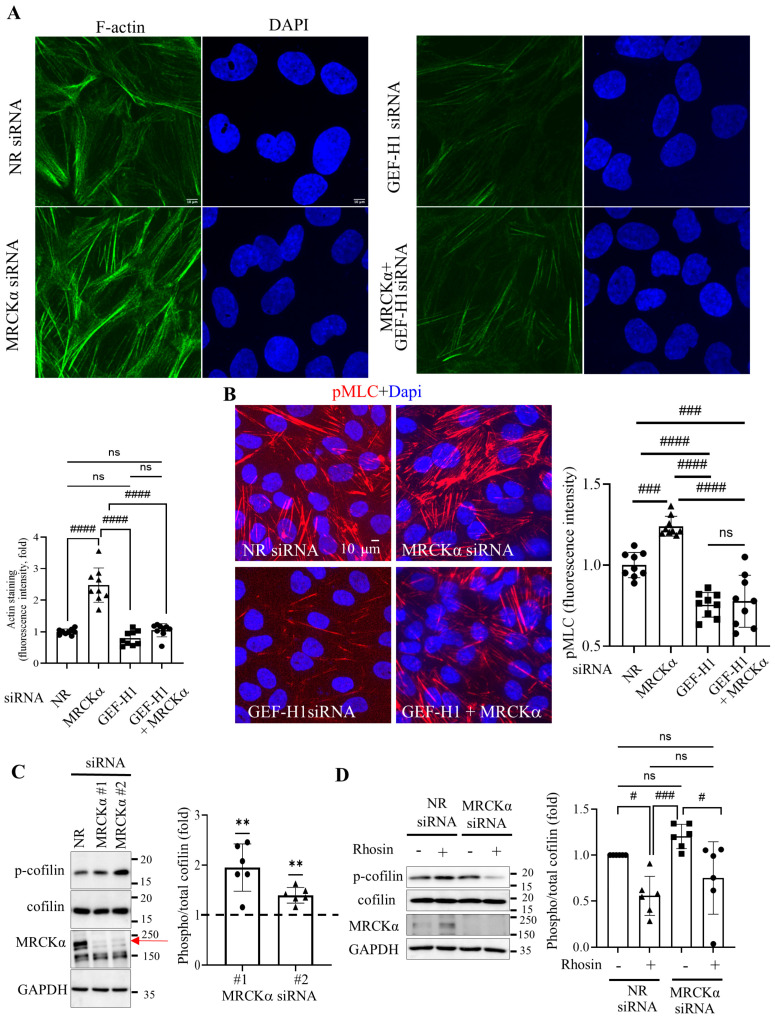
MRCKα reduces GEF-H1-dependent actin stress fibers and phospho-MLC. RPTEC/hTERT cells were transfected with GEF-H1 siRNA and MRCKα siRNA #1 for 48 h. (**A**,**B**) F-actin was visualized using Alexa Fluor^®^ 488 Phalloidin (**A**), and phospho-Ser18/Thr19 MLC was stained using an antibody (**B**). Nuclei were counterstained with DAPI. Pictures were taken using a WaveFX spinning-disk confocal microscope (63× magnification). The scale bar represents 10 µm. The graphs show fluorescence intensity for F-actin staining (**A**) or pMLC intensity (**B**) in *n* = 9 fields from 3 experiments. ns: non-significant; ### *p* < 0.001; #### *p* < 0.0001 (one-way Anova). (**C**). Phosphorylated and total cofilin in LLC-PK_1_ cells transfected with the indicated siRNAs were quantified using Western blotting. Phospho-cofilin was normalized using the corresponding total cofilin and expressed as fold change from control (taken as 1). The red arrow points to MRCKα on the blot. For each siRNA *n* = 6 ** *p* < 0.01 vs. control (Welch *t*-test). (**D**) LLC-PK_1_ cells were transfected with NR or MRCKα siRNA #1. After 48 h the cells were treated with Rhosin for 30 min (30 µM), then phospho- and total cofilin were detected and quantified as in (**B**). The graph shows data from *n* = 6 experiments. # *p* < 0.05, ### *p* < 0.001, one way ANOVA with Šídák’s multiple-comparisons test vs. the indicated condition. ns: non-significant.

We next explored the effects of MRCKα on actin remodelling in tubular cells. MRCKα was silenced in RPTEC/hTERT (Figure 3A) or LLC-PK_1_ cells (Figure S2A) with or without co-silencing GEF-H1. Visualizing F-actin using Alexa Fluor^®^ 488 Phalloidin revealed that MRCKα silencing induced in both cell types a general increase in the intensity of F-actin staining. Co-silencing of GEF-H1 potently reduced this effect, showing that in resting tubular cells MRCKα is a constitutive inhibitor of GEF-H1-dependent F-actin formation.

MRCKα was reported to augment MLC phosphorylation [42], thereby increasing its activity. Therefore, we asked whether this direct pro-phosphorylation effect or its effect on suppression of RhoA-dependent MLC phosphorylation [43] dominates in the kidney epithelial cells. We silenced MRCKα and stained pMLC in RPTEC/hTERT (Figure 3B) or LLC-PK_1_ cells (Figure S2B). We found that pMLC was augmented in both cell types following MRCKα silencing. Co-silencing GEF-H1 reduced this effect, showing that MRCKα depletion enhances MLC phosphorylation through GEF-H1, and likely RhoA/Rho kinase (Figure 3B and Figure S2). Another mechanism whereby RhoA could augment stress fibers is through Rho kinase dependent activation of LIM domain kinase (LIMK), that in turn phosphorylates and inactivates the F-actin severing protein cofilin. However, MRCK itself was shown to promote LIMK-dependent cofilin phosphorylation [44]. Therefore, we next assessed the overall effect of MRCKα silencing on cofilin phosphorylation in tubular cells. As shown on Figure 3C, MRCKα depletion using two independent siRNAs significantly increased phospho-cofilin levels. Moreover, this effect was mediated by RhoA, since the RhoA inhibitor Rhosin prevented the MRCKα silencing-induced upregulation in phospho-cofilin (Figure 3D).

### 3.4. MRCKα Exerts a Negative Feedback on TGF β1-Induced GEF-H1 Activation

**Figure 4:**
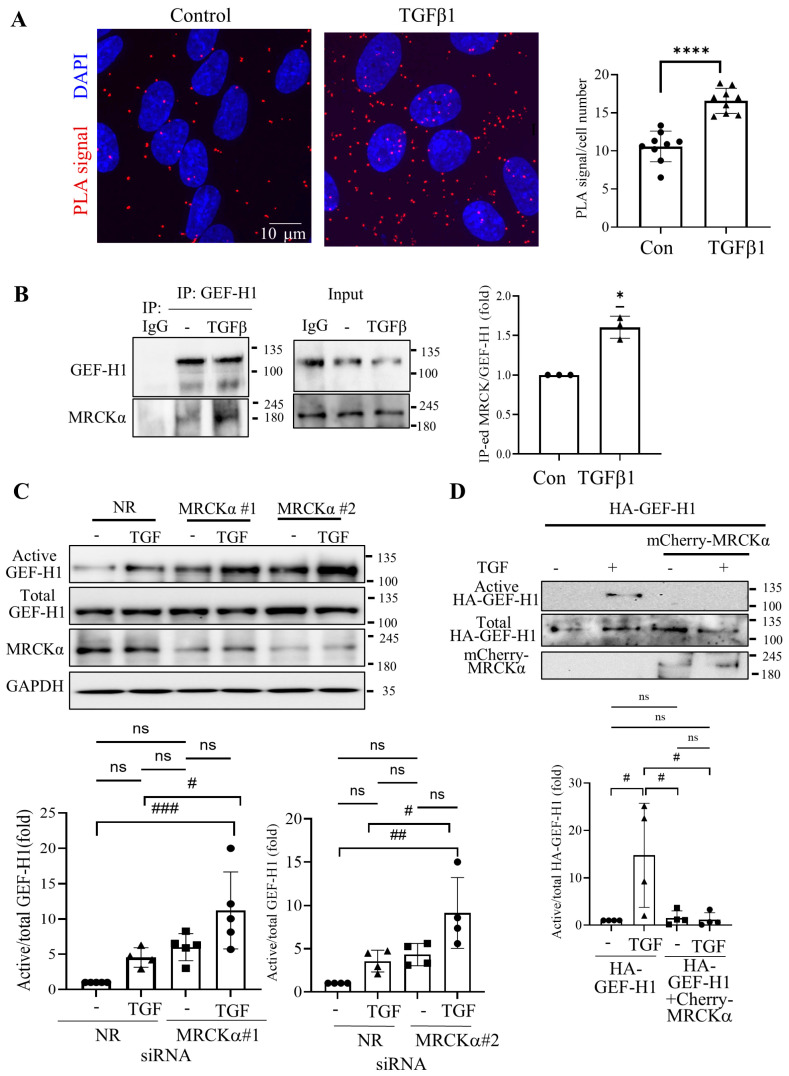
MRCKα blunts GEF-H1 activation induced by TGFβ1. (**A**). RPTEC/hTERTs were stimulated by TGFβ1 (10 ng/mL) for 15 min, and PLA was performed and quantified as in Figure 1 (**** *p* < 0.0001, unpaired *t*-test, *n* = 3 independent experiments, 3 fields/experiment). (**B**). RPTEC/hTERT cells were stimulated by TGFβ1 for 15 min and GEF-H1 IP-ed as in Figure 1. Co-precipitated MRCKα was detected and quantified using Western blotting, co-IP-ed MRCKα was normalized to IP-ed GEF-H1, and expressed as fold change from control. The graph shows *n* = 3, * *p* < 0.05 (Welch’s *t* test). (**C**). LLC-PK_1_ cells were transfected with control (NR) or MRCKα-specific siRNA #1 or #2. Cells were stimulated with TGFβ1 for 15 min and active GEF-H1 precipitated and quantified as in Figure 2 (*n* = 4–5, # *p* < 0.05, ## *p* < 0.01, ### *p* < 0.001, one way ANOVA with Šídák’s multiple-comparisons test vs. the indicated condition. (**D**). LLC-PK_1_ cells were transfected with HA-tagged GEF-H1 with or without mCherry-tagged-MRCKα, and 48 h later the cells were stimulated with TGFβ1 (10 min). GEF-H1 activation was measured as in A, and the precipitation of active HA-GEF-H1 was detected by Western blotting. The precipitated (active) HA-GEF-H1 was normalized to total HA-GEF-H1 (*n* = 4, # *p* < 0.05, one way ANOVA with Šídák’s multiple-comparisons test vs. the indicated condition). ns: non-significant.

GEF-H1 is known to be activated by various cytokines, including TGFβ1 [11,26] that plays a role in fibrogenic epithelial reprogramming. To further understand the role of MRCKα in GEF-H1 regulation, we asked whether TGFβ1 can alter the interaction between MRCKα and GEF-H1. We repeated the PLA in cells that were untreated or stimulated with TGFβ1 (Figure 4A). Interestingly, we found that TGFβ1 significantly increased the number of PLA puncta, suggesting enhanced interaction between the two proteins. To verify this conclusion, we immunoprecipitated GEF-H1 in unstimulated and TGFβ1 stimulated RPTEC/hTERT cells, and found that indeed, TGFβ1 elevated the amount of MRCKα that co-precipitated with GEF-H1 (Figure 4B). Thus, acute treatment with TGFβ1 enhanced the interaction between these two proteins. This led us to hypothesize that in addition to inhibiting basal GEF-H1, MRCKα might also exert negative feedback that blunts stimulus-induced GEF-H1 activation. To test this assumption, we compared the magnitude of stimulus-induced GEF-H1 activation with or without silencing MRCKα in LLC-PK_1_ cells. In accordance with previously published data, TGFβ1 induced a well detectable activation of GEF-H1 (Figure 4C). As shown earlier (Figure 2), MRCKα silencing using two independent siRNAs augmented basal GEF-H1 activity. Importantly, TGFβ1 induced significantly stronger GEF-H1 activation when MRCKα was silenced, supporting the existence of a negative feed-back loop (Figure 4C).

To substantiate our conclusion that MRCKα can suppress GEF-H1 activation, we asked whether MRCKα overexpression can affect GEF-H1 activation. To this end, we overexpressed an mCherry-tagged MRCKα in LLC-PK_1_ cells along with an HA-tagged GEF-H1. This allowed us to follow activation of HA-GEF-H1 selectively in cells that also overexpressed mCherry-MRCKα. Activity of HA-GEF-H1 was followed using the RhoA(G17A) precipitation assay. As shown on Figure 4D, HA-tagged GEF-H1 was strongly activated by TGFβ1. In contrast, when MRCKα was also expressed along with HA-GEF-H1, TGFβ1-induced GEF-H1 activation was abolished. Taken together, these data substantiate that MRCKα can suppress stimulus-induced GEF-H1 activation.

### 3.5. MRCKα Silencing Elevates Expression of Fibrosis-Related Genes

**Figure 5:**
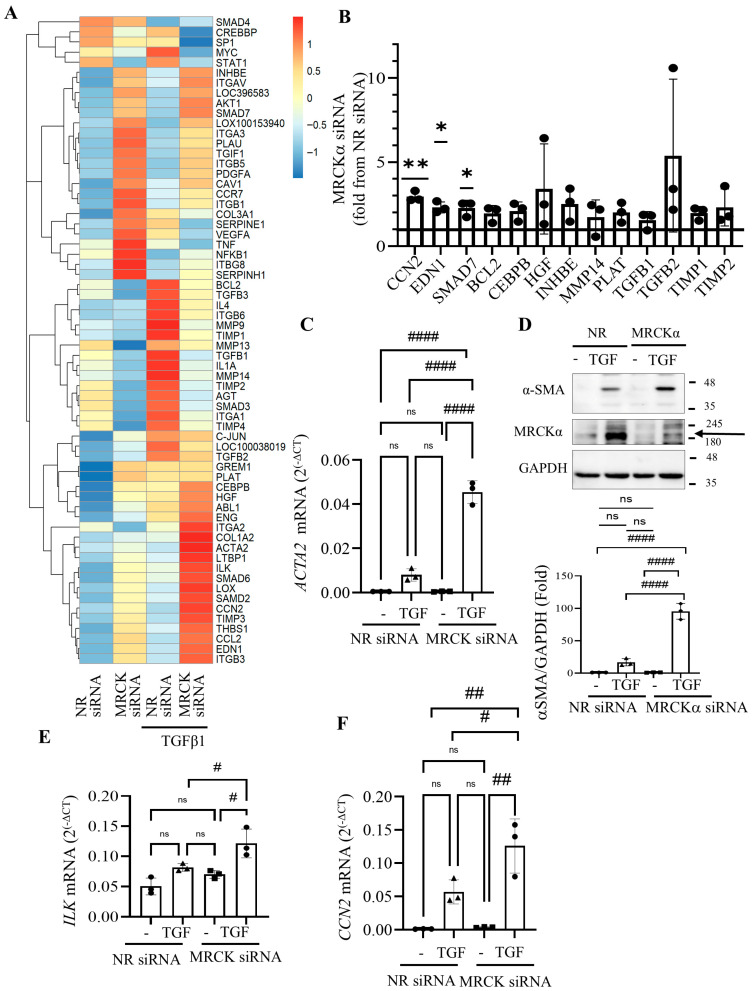
MRCKα suppresses several fibrosis-associated genes. (**A**). LLC-PK_1_ cells were transfected with NR or MRCKα specific siRNA for 48 h. Twenty-four h post-transfection the medium was replaced with serum-free DMEM supplemented with 10 ng/mL TGFβ1 for 24 h, as indicated. The samples were analyzed using a Qiagen RT^2^ Profiler™ Pig Fibrosis gene PCR array. The heatmap shows Z scores, calculated using fold changes of the relative gene expression. (**B**) Genes that are upregulated in the array by at least 1.5-fold upon MRCKα silencing compared to the control (taken as 1). *n* = 3, * *p* < 0.05, ** *p* < 0.01, Welch’s *t* test. (**C**) Data from the fibrosis array for the *ACTA2* mRNA (*n* = 3, #### *p* < 0.0001, one-way Anova vs. the indicated condition). (**D**) LLC-PK_1_ cells were transfected with the indicated siRNAs, and 4 h later the medium was changed to serum-free DMEM with or without TGFβ1 (10 ng/mL) for 48 h. αSMA, MRCKα and GAPDH were detected by Western blotting (*n* = 3, ns: non-significant; #### *p* < 0.0001, one way ANOVA with Šídák’s multiple-comparisons test vs. the indicated condition). The MRCKα specific band is indicated by the arrow. (**E**,**F**) Data from the fibrosis array for the *ILK* (**E**) and *CCN2* (**F**) mRNA (*n* = 3, # *p* < 0.01, ## *p* < 0.01, one-way Anova vs. the indicated condition). Note, that in (**F**) using unpaired *t*-test the difference is significant between control and siMRCK as well, but due to the robust increase under TGFβ conditions, the significance disappears when ANOVA is used. ns: non-significant.

Studies from us and others have implicated GEF-H1/RhoA signalling in pro-fibrotic epithelial reprogramming [6,23,24]. Since our above-described findings indicated that MRCKα was an inhibitor of GEF-H1, we next asked if MRCKα also controlled expression of key pro-fibrotic genes in tubular cells. To address this, we used a Qiagen RT^2^ Profiler™ Pig Fibrosis PCR Array allowing assessment of the expression of 84 genes. LLC-PK_1_ cells were transfected with control (NR) or MRCKα-specific siRNA followed by treatment with or without TGFβ1. Figure 5A depicts results from the array expressed as Z scores, revealing differences from the mean calculated for each gene using all treatment groups. We noted upregulation of several genes upon MRCKα silencing. Figure 5B depicts genes that were at least 1.5 fold elevated upon MRCKα silencing, including *CCN2*/CTGF, endothelin-1 (*EDN1*) and *SMAD7* (see also Figure S3A,B). Other genes showing a consistent increasing trend include the TGF superfamily members TGFβ1 and 2 (*TGFB1*, *TGFB2)*, and inhibin β (*INHBE*); ECM regulating proteins *TIMP1* and 2, and matrix metalloproteinase 14 (*MMP14*); the pro-survival protein *BCL2*, as well as Tissue-type plasminogen activator (*PLAT*). For these genes the fold changes varied in a gene-dependent manner between 1.3 and 10-fold. Importantly, many of the upregulated genes have been implicated in fibrosis.

### 3.6. MRCKα Silencing Promotes MRTF-Dependent Gene Transcription

Several of the genes explored were also induced by TGFβ1, a key pro-fibrotic cytokine. Given that MRCKα silencing augmented the effects of TGFβ1 on RhoA, we next identified the set of genes that are known to be affected by RhoA signalling. Indeed, several RhoA-dependent genes were affected by MRCKα and exhibited stronger upregulation when both MRCKα silencing and TGFβ1 were applied. As shown on Figure 5C, *ACTA2* (encoding α smooth muscle actin, α-SMA), a hallmark of the myofibroblast phenotype, and a RhoA activated gene [6,45] was marginally upregulated by MRCKα silencing. In contrast, TGFβ1 caused about 16-fold upregulation. Importantly, MRCKα silencing significantly and substantially augmented the effect of TGFβ1 on *ACTA2*. In fact, the combined treatment of MRCKα silencing and TGFβ1 elevated *ACTA2* close to a 100-fold compared to the control (Figure 5C). Western blotting verified that the effect of TGFβ1 on the α-SMA protein was strongly augmented by MRCKα depletion (Figure 5D). MRCKα silencing also signifcantly increased the effect of TGFβ1 on integrin linked protein kinase (*ILK*) and *CCN2* (Figure 5E,F). Finally, a similar, albeit much weaker pattern was also seen in some other genes including EDN1 and SMAD7 (Figure S3). Taken together, these experiments revealed that MRCKα suppressed the basal expression of a variety of Rho-regulated genes involved in fibrosis and mitigated TGFβ1-induced gene expression.

**Figure 6:**
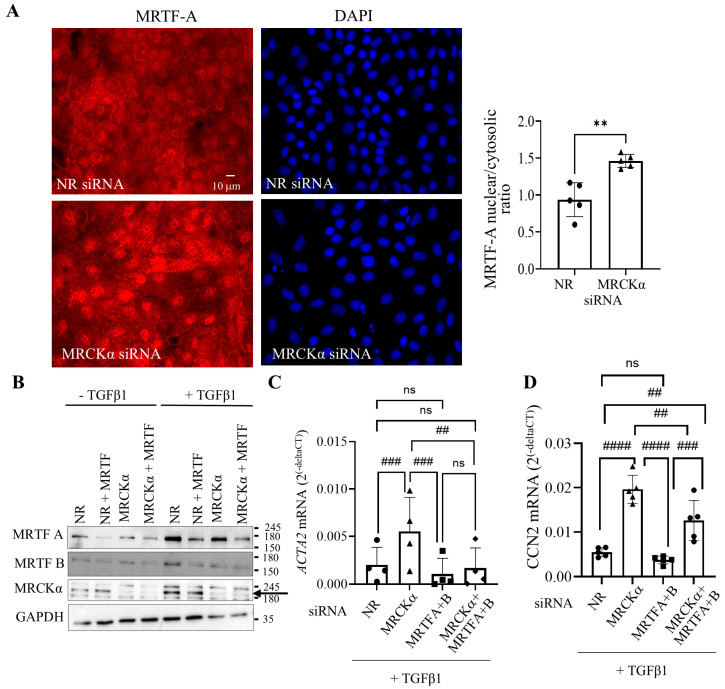
MRCKα regulates MRTF-A nuclear translocation and MRTF-dependent gene transcription. (**A**). LLC-PK_1_ cells grown on coverslips were transfected with the indicated siRNAs, and 32 h later the medium was changed to serum-free DMEM for 16 h. MRTF-A was visualized by immunofluorescence using an Alexa Fluor 555-labelled secondary antibody. Nuclei were stained using DAPI. Pictures were taken using a Zeiss Widefield Microscope (63× objective). The scale bar represents 10 µm. The graph shows quantification of the nuclear/cytosolic ratio. ** *p* < 0.01 (unpaired *t*-test, *n* = 5 fields). (**B**). LLC-PK_1_ cells were transfected and treated with TGFβ1 (48 h), and the indicated siRNAs, and proteins detected using the indicated antibodies. The arrow points to MRCKα. (**C**,**D**). LLC-PK_1_ cells were transfected with the indicated siRNAs, and where indicated, treated with TGFβ1 for 24 h. RT-PCR was performed to measure *ACTA2* (**C**) and *CCN2* (**D**) mRNA as described in the Methods. In (**C**) changes were expressed as fold change from NR siRNA transfected samples taken as 1 (*n* = 4 (**C**) and *n* = 5 (**D**), ns: non-significant; ## *p* < 0.01, ### *p* < 0.001, #### *p* < 0.0001, one-way Anova vs. the indicated condition).

MRTF-A and B are crucial fibrogenic transcription factors activated by RhoA signalling [14]. In fact, several of the genes we found to be augmented by MRCKα silencing are known RhoA-MRTF targets, suggesting that MRCKα might regulate MRTF. To test this assumption, we silenced MRCKα in LLC-PK_1_ cells, and explored effects on MRTF in the absence or presence of TGFβ1. Immunofluorescent staining visualizing MRTF-A (Figure 6A) revealed that in control cells MRTF-A was cytosolic or partially nuclear. MRCKα depletion by itself augmented nuclear presence of MRTF-A. Thus, MRCKα indeed acts as a suppressor of MRTF nuclear accumulation. To explore the functional relevance of MRTF regulation by MRCKα, we depleted this protein with and without co-silencing of the two MRTF isoforms, MRTF-A and B. Figure 6B verifies successful downregulation of all three proteins. *ACTA2* and *CCN2* are typical MRTF-regulated pro-fibrotic genes [6,17,46]. RT-qPCR analysis verified that MRTF-A and B silencing strongly and significantly reduced the upregulation of *ACTA2* (Figure 6C) and *CCN2* (Figure 6D) induced by the combination of MRCKα siRNA and TGFβ1. MRTFA and B depletion also reduced the effects of MRCKα alone on *CCN2* (Figure S3C,D), albeit the inhibition by MRTF-α and B depletion did not reach significance. Overall, our data strongly support an anti-fibrogenic effect for MRCKα through suppressing MRTF-dependent gene transcription.

### 3.7. MRCKα Expression Is Reduced in a Mouse Kidney Fibrosis Model

**Figure 7:**
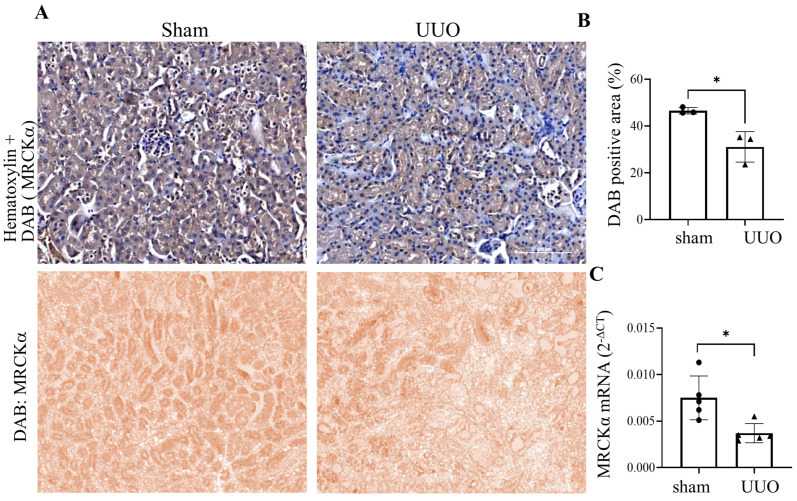
MRCKα protein and mRNA expression are reduced in UUO kidneys. (**A**) (**Top**): Representative IHC staining of MRCKα (brown) in kidney sections from sham (**left**) and UUO (7 day, **right**) mice against hematoxylin counterstain (blue). Sections were visualized with DAB (brown) to detect MRCKα and stained with hematoxylin (blue) for nuclei. Scale bar: 100 µm. (**Bottom**): Isolated DAB channel from H-DAB deconvolution of selected representative IHC images in sham and UUO kidney sections. The images were exported from a total slide scan (see Section 2.12). (**B**) Quantification of kidney MRCKα staining (unpaired *t*-test, *n* = 3 mice, mean of 4–5 ROIs/kidneys * *p* < 0.05, unpaired *t*-test. (**C**) RT-qPCR analysis showed of sham and UUO 7 day kidneys (*n* = 5, unpaired *t*-test * *p* < 0.05).

To gain insight into possible changes to MRCKα induced by fibrosis, we explored its expression in kidney samples from the UUO mouse model of kidney injury and fibrosis. In this widely used model ligation of one ureter increases the pressure in the tubules, ultimately leading to robust interstitial fibrosis by day 7 [6,47]. We have previously shown that RhoA activity increased in the kidneys as soon as day 1 after UUO surgery [6], and GEF-H1 was elevated by day 7 [11]. Moreover, in the UUO kidneys, MRTF-dependent genes, such as *ACTA2* and *CCN2* were strongly upregulated [6]. Therefore, we asked whether MRCKα expression was also altered in the UUO kidneys. As shown on Figure 7A,B, MRCKα was well detectable in kidney tubules in the sham samples. UUO at day 7 caused a significant loss of MRCKα staining in the tubules. Further, RT-PCR revealed a significant decrease in the whole kidney mRNA of MRCKα (Figure 7C). Taken together, these data suggest that the onset of fibrosis is associated with a reduction in MRCKα.

## 4. Discussion

The key finding of this study is the demonstration that MRCKα interacts with and inhibits GEF-H1. We showed that MRCKα depletion augmented both basal and TGFβ1-induced GEF-H1 activity, promoted RhoA and MRTF activation and augmented TGFβ1-induced expression of several RhoA-regulated fibrosis-related genes. Finally, we also found reduced expression of MRCKα in a murine kidney fibrosis model. Taken together, our study supports a role for MRCKα as a new suppressor of the pro-fibrotic epithelial phenotype switch.

GEF-H1 is a ubiquitously expressed exchange factor for RhoA, RhoB and Rac, with vital roles in cytoskeletal organization, motility, epithelial polarization and permeability, as well as gene expression, including pro-fibrotic epithelial reprogramming (reviewed in [21,22]). Fine regulation of this protein is crucial for normal cellular functions, including epithelial homeostasis. Accordingly, GEF-H1 dysregulation was associated with various diseases. For example, GEF-H1-dependent signalling is upregulated in various cancers (e.g., [48,49,50,51,52]). Studies from us and others also implicated GEF-H1 in heart, kidney and eye disease and organ fibrosis [11,23,25,26]. These diverse physiological and pathological functions are fine-tuned in a complex and incompletely understood manner. Resting GEF-H1 activity is suppressed by binding to intercellular junction proteins and/or the microtubules [12,27,28,29,53,54,55]. Multiple phosphorylation sites in GEF-H1 that are targeted by a variety of kinases are also crucial for tight spatiotemporal control [21]. For example, ERK1/2 is an activating kinase that was implicated in mediating effects of an array of stimuli (e.g., [13,37]). Interestingly, however, the majority of kinases that were shown to interact with GEF-H1 appear to be inhibitory. Examples include the PAK family [29,56], Protein kinase A [57], MARK2/Par1b [30,58] and cell cycle associated kinases such as Aurora A [59]. Thus, kinase-dependent suppression is a key mechanism of GEF-H1 activity control.

Our current study provides characterization of the effects of MRCKα as an inhibitor of GEF-H1. Using co-precipitation and in situ proximity ligation we verified interaction between MRCKα and GEF-H1 in kidney tubular cells. This finding is in line with a previous study that found these proteins in complex with BepC, an effector protein of the bacterium Bartonella [41]. Nonetheless, the functional relevance of this interaction remained unclear; e.g., MRCKα knockout did not affect BepC-induced cytoskeleton remodelling. Our study shows that MRCKα silencing augments GEF-H1 activity in tubular cells, indicating that this interaction is inhibitory. We also showed that MRCKα binding did not require the C-terminal portion of GEF-H1. Since the N-terminus of the protein contains the DH domain that physically interacts with RhoA, and harbors the GEF activity, one possibility is that MRCKα binding might mask the site for the GEF-H1-RhoA interaction, resulting in inhibition of the effect of GEF-H1 on RhoA. MRCKα may also be in a tripartite complex with GEF-H1 and RhoA, affecting RhoA activation. Alternatively, MRCKα may phosphorylate GEF-H1, leading to its suppression. Although in LLC-PK_1_ cells we have not been able to detect consistent changes in GEF-H1 phosphorylation upon MRCKα silencing, this may be due to technical limitations. Thus, further studies are warranted to define the exact mechanism, which may include MRCK-induced inhibitory GEF-H1 phosphorylation and/or hindrance of RhoA association or activation by GEF-H1.

Surprisingly, TGFβ1, the chief fibrogenic cytokine, which is known to activate GEF-H1 [13,26], increased the PLA signal and the co-precipitation between GEF-H1 and MRCKα. Thus, a larger pool of MRCKα and GEF-H1 was interacting following acute stimulation, raising the possibility of a negative feedback mechanism that may limit GEF-H1 activity, and prevent its overactivation. This conclusion is supported by our data showing that MRCKα silencing augments not only basal, but also stimulus-induced GEF-H1 activation. Since GEF-H1 is activated by both MRCKα silencing and by TGFβ1, and MRCKα downregulation activates RhoA through GEF-H1, it is very likely that RhoA activation by the combination of MRCKα silencing and TGFβ1 is GEF-H1-dependent; albeit this possibility awaits direct verification. Nonetheless, as strong support of this conclusion, we found that overexpression of a tagged MRCKα mitigates TGFβ1-induced GEF-H1 activation.

Members of the MRCK family were identified as interactors of the Rho family small GTPase Cdc42 [36,42]. However, the exact role of Cdc42 in MRCK regulation remains unclear. Overexpression of active Cdc42 or silencing endogenous Cdc42 did not result in MRCKα activity changes [60,61]. Instead, Cdc42 binding likely controls MRCK localization, as Cdc42 downregulation was found to cause loss of lamellipodial MRCK in HeLa cells [61]. On the other hand, expression of a kinase-dead MRCKα was shown to block some downstream effects of active Cdc42 [42]. Localized crosstalk between Rho family proteins has been documented in many systems, and MRCKα may represent a focal point for such interplay. Our finding that MRCKα is an inhibitor of GEF-H1/RhoA reveals an additional layer of complexity whereby Cdc42 might affect localized RhoA activity. Cdc42-dependent recruitment of MRCKα may lead to local inhibition of GEF-H1/RhoA signalling, for example in the lamellipodium of migrating cells, or during the development of epithelial polarity. Such crosstalk could also fine-tune perijunctional actomyosin contractility [62]. Interestingly, GEF-H1 appears to integrate input from Rac and Cdc42 through PAK family kinases that are effectors of these small GTPases. PAK1 was shown to phosphorylate and inactivate GEF-H1, at least in part by regulating its microtubule binding [29]. Further, in tubular cells we showed that TNFα-induced Rac activation promoted ERK-dependent stimulation of GEF-H1 towards RhoA [63], and GEF-H1 phosphorylation status can determine which small GTPAse (e.g Rac or RhoA) is activated downstream [63]. Together with the current study, these data further highlight the complex crosstalk between various small GTPases and their regulators.

One key effect of MRCKα is the regulation of acto-myosin contractility. MRCKs can directly phosphorylate MLC, and can also control cofilin, a ubiquitous actin severing protein, by activating LIMK [44,61,64,65,66]. LIMK phosphorylates, and thereby inactivates cofilin, leading to increased actin polymerization [67]. We found that in tubular cells, downregulating MRCKα augmented F-actin levels and increased pMLC through GEF-H1. Further, silencing MRCKα induced cofilin phosphorylation. The observations that MRCKα silencing elevates pMLC, actin and phospho-cofilin are in apparent contradiction with findings that show that MRCKα elevates pMLC and activates LIM kinase [44,61,64,65,66]. However, both LIMK and MLC activity are also controlled by RhoA/Rho kinase, and the effects of MRCKα through these pathways appear to be opposite. Thus, the overall effect of MRCKα activity on pMLC, cofilin and F-actin in a given cell likely depends on the balance of the direct activating and indirect inactivating (through RhoA/Rho kinase) effects. In line with our findings, an increase in phospho-cofilin was also observed in breast cancer cells after MRCKα knockout [68]. Overall, the magnitude of MLC and cofilin activating effects of MRCKα might be cell type-dependent, and are balanced by its suppressor effects on RhoA. In tubular cells the RhoA activating effect of reduced MRCKα appears to supersede the more direct effects of MRCKα on MLC and cofilin. This conclusion is also supported by the facts that (1) MRCKα-induced pMLC and F-actin increase are mitigated when GEF-H1 is also silenced; and (2) RhoA inhibition prevents the MRCKα silencing-induced cofilin phosphorylation. Overall the cytoskeletal effects we found in tubular cells are in line with the predominance of a suppressor effect of MRCKα on RhoA-dependent cytoskeleton remodelling.

GEF-H1 and RhoA are also key regulators of injury-induced epithelial reprogramming in tubular cells, and promote the production of pro-fibrotic mediators [6]. Reprogrammed epithelial cells release various mediators to activate mesenchymal cells and enhance ECM production, a key step in fibrosis. Indeed, our data show that MRCKα silencing augments the production of the matricellular signaling protein CCN2 (CTGF), a key profibrotic mediator. MRCKα silencing by itself also augments other fibrosis-related genes, and promotes the effects of TGFβ1. We also showed that MRCKα altered MRTF nuclear translocation, and MRTF-dependent gene transcription (e.g., *ACTA2* and *CCN2*) [6]. However, the overall effect of MRCKα silencing is complex. While it exerted a general positive effect on many genes we tested, a subset of genes showed reduced expression upon MRCKα silencing. Thus MRCKα may be a positive regulator of some genes. Further, MRCKα silencing also significantly augmented *SMAD7*, that exerts a negative feed-back on TGFβ signalling and was found to have an anti-fibrotic role [69]. Thus, the comprehensive effect of MRCKα on TGFβ1 signalling warrants further studies.

Finally, we also provide evidence that MRCKα expression is reduced in the UUO murine kidney fibrosis model. The decrease in total mRNA is in line with reduced tubular expression, since up to 80% of the kidney tissue consists of tubules. Indeed, IHC showed significantly reduced tubular staining in the UUO kidneys. Our previous studies have shown that in the UUO kidneys MRTF was activated and MRTF-dependent genes, including *ACTA2* and *CCN2* were upregulated [6]. Thus, our current findings suggest that reduced MRCKα expression in the fibrotic kidneys may contribute to the activation of MRTF and the upregulation of crucial MRTF-dependent genes. Overall, our current study supports the hypothesis that loss of MRCKα as a RhoA suppressor might contribute to epithelial-mesenchymal crosstalk during the development of kidney fibrosis. Future studies involving animal models should reveal the overall role of MRCKα in fibrogenesis.

## 5. Conclusions

This study identified a new negative regulatory mechanism controlling GEF-H1/RhoA signalling under basal and cytokine-stimulated conditions. The MRCKα-GEF-H1 interaction modulates actin remodelling in tubular cells. Accordingly, MRCKα is a novel regulator of MRTF signalling, that plays a crucial role in fibrogenic tubular reprogramming. Taken together, our study implicates MRCKα as a new suppressor of fibrosis.

## Figures and Tables

**Table 1:** Short interfering RNA sequences against porcine proteins (LLC-PK_1_ cells).

Target	siRNA Sequence
Porcine MRCKα #1	GGG AAA UGA AGA AGG GUU AUU
Porcine MRCKα #2	AGU UAG AAG AAG AGG UAA AUU
Porcine MRTF-A	CCA AGG AGC UGA AGC CAA A
Porcine MRTF-B	CGA CAA ACA CCG UAG CAA A
Porcine GEF-H1	CAAGAGCAUCACAGCCAAG

**Table 2:** Primers for detecting porcine mRNA.

Gene	Sequence
*CCN2*/CTGF F	GTG AAG ACA TAC CGG GCT AAG
*CCN2*/CTGF R	GAC ACT TGA ACT CCA CAG GAA
*ACTA2* F	CGTCCTAGACATCAGGGGGT
*ACTA2* R	GGGGCAACACGAAGCTCATT
*PPIA* F	CGG GTC CTG GCA TCT TGT
*PPIA* R	TGG CAG TGC AAA TGA AAA ACT G

**Table 3:** Primers for detecting murine mRNA.

Gene	Sequence
*CDC42BPA* F	TCGAAGCAAACATGTCCGGA
*CDC42BPA* R	CGTCTCCACACTGAAGCACT
*GAPDH* F	CATCACTGCCACCCAGAAGACTG
*GAPDH* F	ATGCCAGTGAGCTTCCCGTTCAG

## Data Availability

The original contributions presented in this study are included in the article/Supplementary Material. Further inquiries can be directed to the corresponding author.

## Supplementary Materials

The following supporting information can be downloaded at: https://www.mdpi.com/article/10.3390/cells15050447/s1. Figure S1: Controls for the proximity ligation assay. RPTEC/hTERT cells were transfected with MRCKα or GEF-H1 siRNA and PLA was performed. Where indicated, the primary antibodies were omitted. The red puncta in 3–5 visual fields/coverslips were counted and divided by the number of nuclei. The graph shows mean +/− SD of 10 fields from 2 independent experiments (*** *p* < 0.001, unpaired *t*-test, *n* = 10). The blots show verification of the downregulation. Figure S2: MRCKα silencing increases F-actin and phospho-MLC is LLC-PK_1_ cells. LLC-PK_1_ cells were transfected with the indicated siRNAs. (**A**) F-actin was visualized using Alexa 488-labelled phalloidin. The blot verifies downregulation. The graph shows quantification of phalloidin staining intensity. (**B**). Phospho-MLC was stained and quantified as described in the methods. Figure S3: Changes in fibrogenic genes induced by MRCKα silencing and TGFβ1 treatment. (**A**,**B**). Data from the mRNA array. (**C**,**D**). Data obtained by RT-PCR in LLC-PK_1_ cells. MRTF-A and B were silenced along with MRCKα and TGFβ1 treatment, as in Figure 6.

## Abbreviations

The following abbreviations are used in this manuscript:

CTGF	Connective tissue growth factor
CCN2	Cellular Communication Network Factor 2
GEF	Guanine Nucleotide Exchange Factor
CKD	Chronic kidney disease
ECM	Extracellular matrix
LIMK	LIM domain kinase
MRCK	Myotonic Dystrophy Kinase-related Cdc42-binding kinase
MRTF	Myocardin-Related Transcription Factor
NR	non-related
PLA	Proximity Ligation Assay
SMA	Smooth muscle actin
TGFβ1	Transforming Growth Factor β1
