# Supplementary figures and images for: MRCKα Is a Suppressor of GEF-H1/RhoA/MRTF Signaling in Tubular Cells
Source: Cells. 2026 Mar 2;15(5):447. doi: 10.3390/cells15050447 (PMC12984664; doi:10.3390/cells15050447)

Fig S1

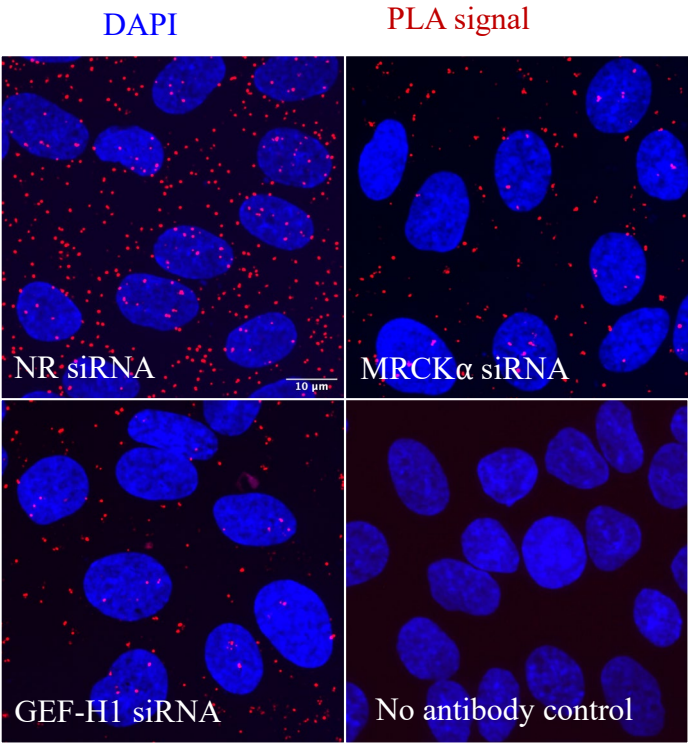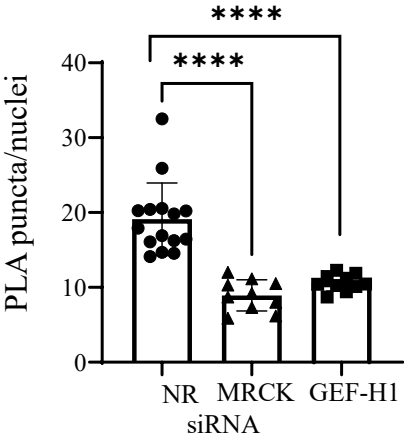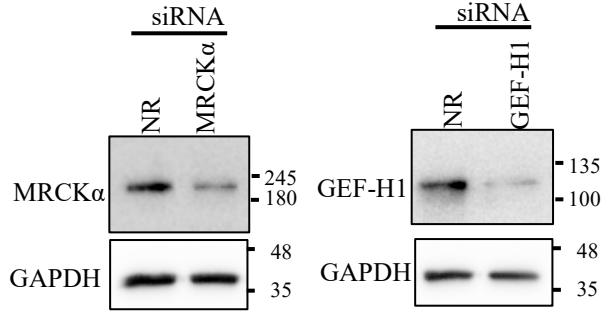

**Fig S2**

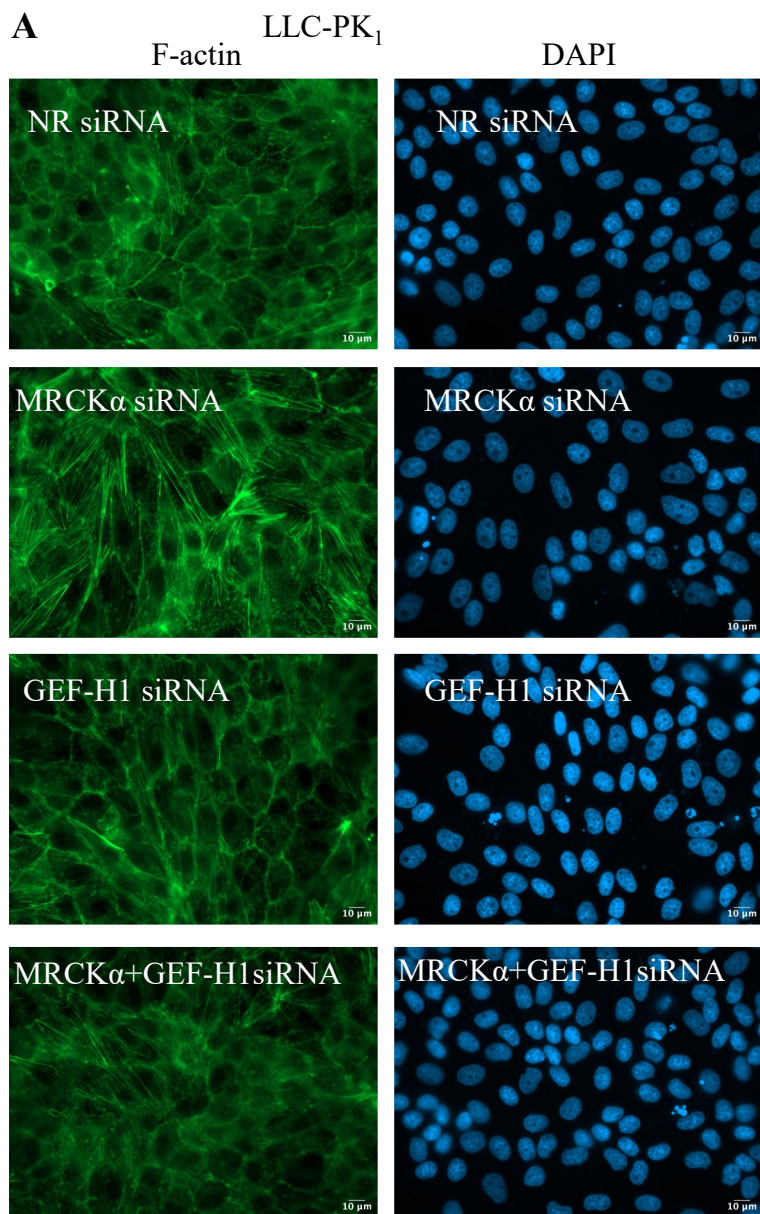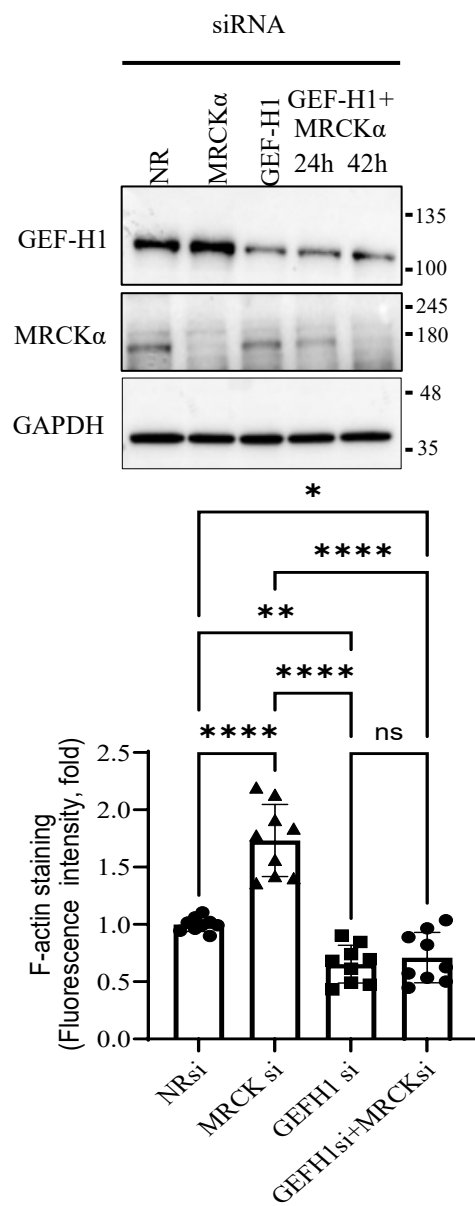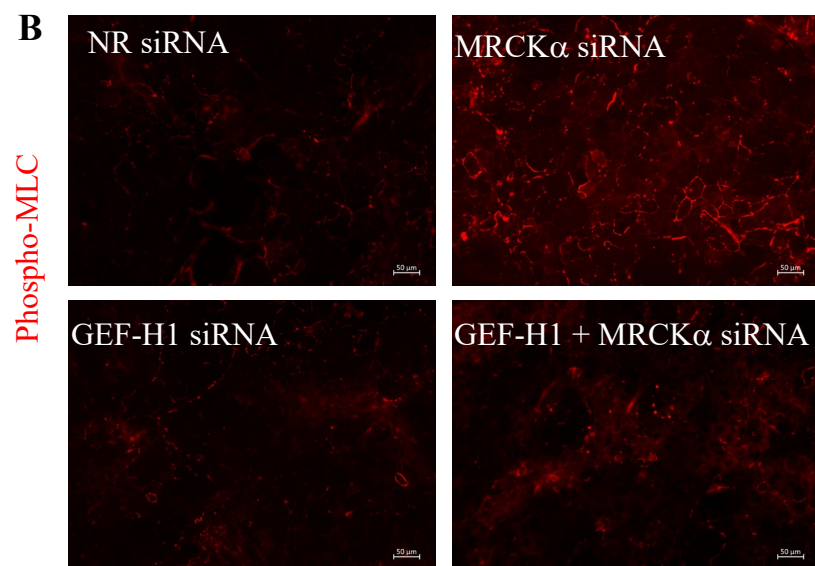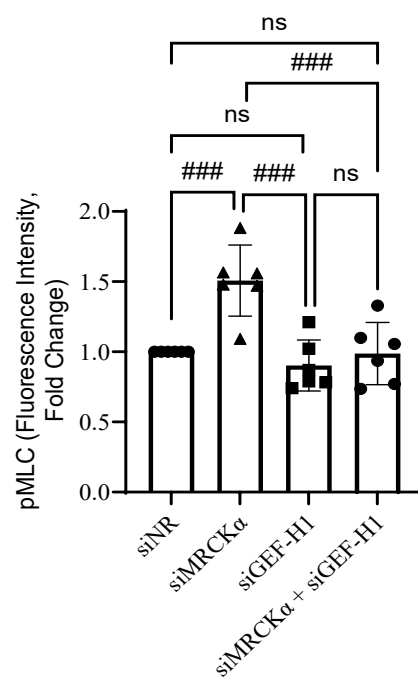

Fig S3

A

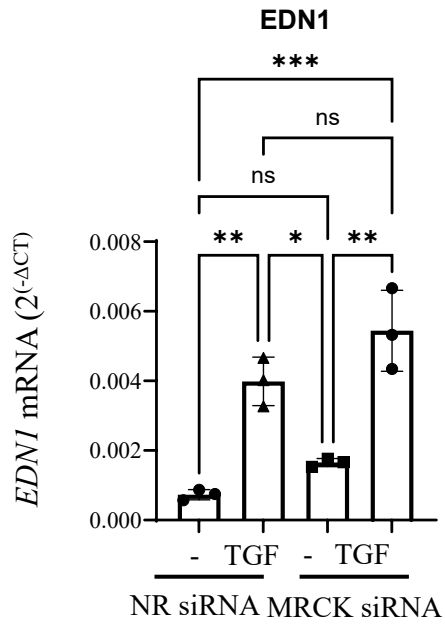

# B

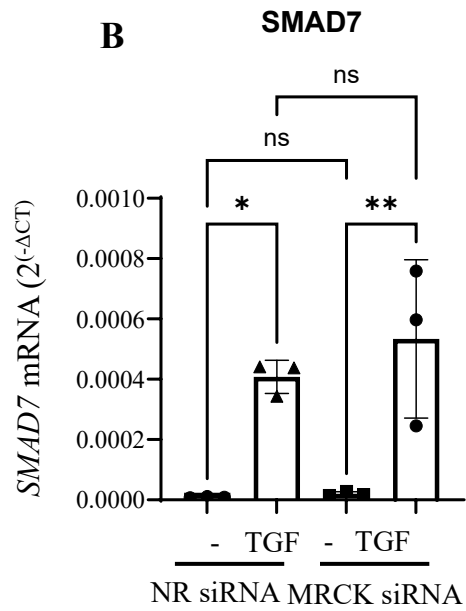

C

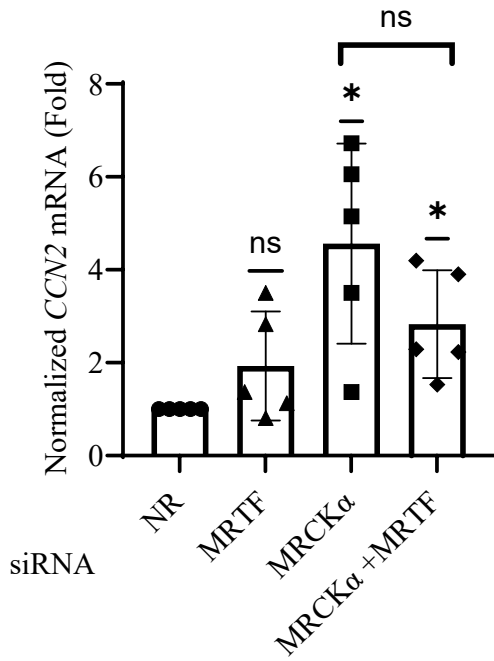

D

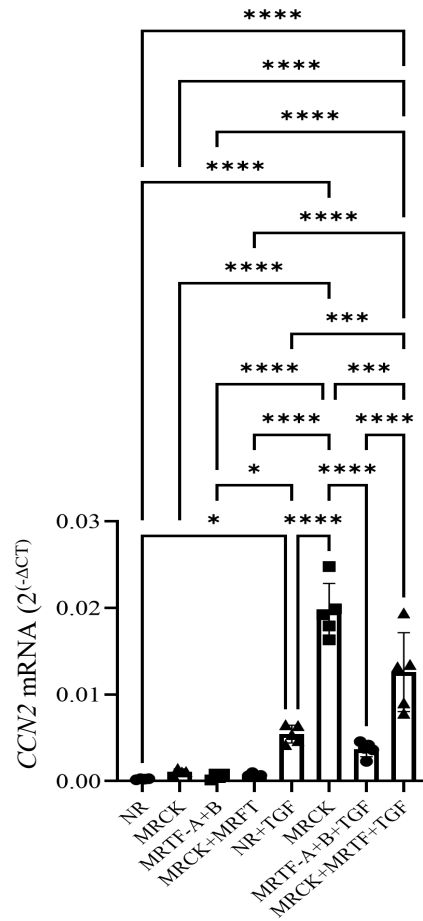

Supplement: Supplementary file 1 [file cells-15-00447-s001.zip › cells-4060003-supplementary.pdf]
